# Solidification Microstructures of the Ingots Obtained by Arc Melting and Cold Crucible Levitation Melting in TiNbTaZr Medium-Entropy Alloy and TiNbTaZrX (X = V, Mo, W) High-Entropy Alloys

**DOI:** 10.3390/e21050483

**Published:** 2019-05-10

**Authors:** Takeshi Nagase, Kiyoshi Mizuuchi, Takayoshi Nakano

**Affiliations:** 1 Research Center for Ultra-High Voltage Electron Microscopy, Osaka University, 7-1 Mihogaoka, Ibaraki, Osaka 567-0047, Japan; 2 Osaka Research Institute of Industrial Science and Technology, 1-6-50 Morinomiya, Joto-ku, Osaka 536-8553, Japan; 3 Division of Materials and Manufacturing Science, Graduate School of Engineering, Osaka University, 2-1 Yamadaoka, Suita, Osaka 565-0871, Japan

**Keywords:** high-entropy alloy, biomaterials, arc melting, levitation melting, microstructure, solidification, thermodynamic calculation

## Abstract

The solidification microstructures of the TiNbTaZr medium-entropy alloy and TiNbTaZrX (X = V, Mo, and W) high-entropy alloys (HEAs), including the TiNbTaZrMo bio-HEA, were investigated. Equiaxed dendrite structures were observed in the ingots that were prepared by arc melting, regardless of the position of the ingots and the alloy system. In addition, no significant difference in the solidification microstructure was observed in TiZrNbTaMo bio-HEAs between the arc-melted (AM) ingots and cold crucible levitation melted (CCLM) ingots. A cold shut was observed in the AM ingots, but not in the CCLM ingots. The interdendrite regions tended to be enriched in Ti and Zr in the TiNbTaZr MEA and TiNbTaZrX (X = V, Mo, and W) HEAs. The distribution coefficients during solidification, which were estimated by thermodynamic calculations, could explain the distribution of the constituent elements in the dendrite and interdendrite regions. The thermodynamic calculations indicated that an increase in the concentration of the low melting-temperature V (2183 K) leads to a monotonic decrease in the liquidus temperature (*T*_L_), and that increases in the concentration of high melting-temperature Mo (2896 K) and W (3695 K) lead to a monotonic increase in *T*_L_ in TiNbTaZrX*_x_* (X = V, Mo, and W) (*x* =  0 − 2) HEAs.

## 1. Introduction

**Figure 1:**
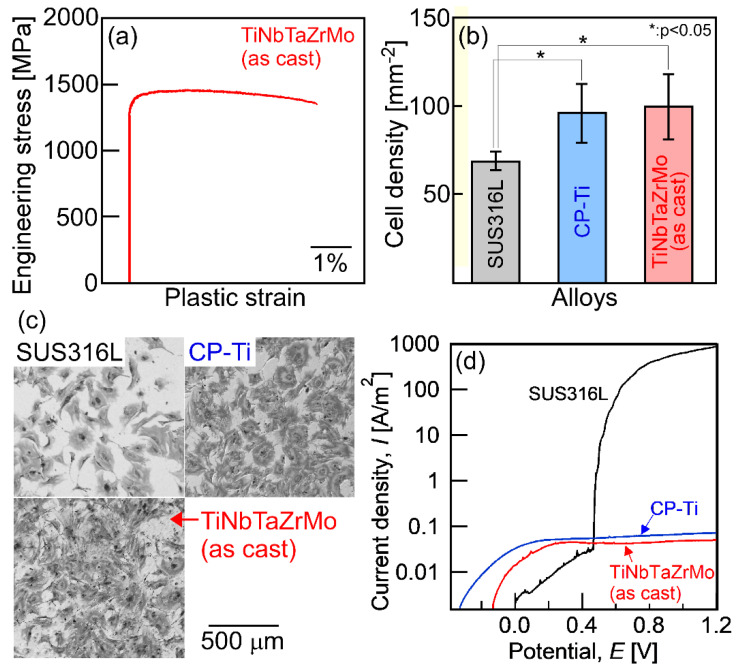
Mechanical properties, biocompatibility, and corrosion resistance of the arc-melted ingots of TiNbTaZrMo bio-high-entropy alloy (HEA). (**a**) Engineering stress–plastic strain compression curve at room temperature [14], (**b**) cell density test results [14], (**c**) Giemsa staining of the osteoblasts culture [14], and (**d**) corrosion test results using normal saline solution at 310 K (37 °C) [18]. SUS316L and CP-Ti mean stainless steel and commercial pure titanium, respectively. The detailed information corresponding to Figure 1a–c can be found in the literature [14], and the corrosion behavior will be reported in a future work [18].

A recently developed concept in the area of multicomponent alloys, which is known as high-entropy alloys (HEAs), has been proposed for use in a new generation of structural and functional metallic materials [1,2,3,4,5,6,7]. Among the various HEAs, those whose constituent elements are group IV elements (Ti, Zr, Hf), group V elements (V, Nb, Ta), and group VI elements (Cr, Mo, Ta) are referred to as refractory HEAs (RHEAs). RHEAs were mainly developed as high-temperature structural materials and/or radiation resistant materials [8,9,10,11,12,13]. Recently, Ti–Nb–Ta–Zr–Mo HEAs, whose constituent elements are group IV, V, and VI elements, were reported as promising metallic biomaterials [14,15,16]. Furthermore, the TiNbTaZr medium-entropy alloy (MEA) was developed as an MEA whose constituent elements are biocompatible [14,17]. In this paper, HEAs and MEAs for metallic biomaterials are denoted as bio-HEAs and bio-MEAs, respectively. Figure 1 shows the typical mechanical properties and the bio-compatibilities of the arc-melted (AM) ingots of the TiNbTaZrMo bio-HEA [15].

Detailed information regarding the engineering stress–plastic strain compression curve (Figure 1a) [14], cell density test results for evaluating the biocompatibility (Figure 1b,c) [14] of stainless steel (SUS316L), commercial pure titanium (CP-Ti), and AM ingots of TiNbTaZrMo bio-HEA are provided in a previous work [14]. Significant differences in biocompatibility between CP-Ti and TiNbTaZrMo bio-HEA AM ingots were not found. The mechanical strength of TiNbTaZrMo bio-HEA AM ingots at room temperature was much higher than that of CP-Ti. Figure 1d shows the corrosion test that was performed using normal saline solution at 310 K (37 °C). The solution was deaerated by bubbling purified nitrogen gas before the corrosion test. The scan rate was 1 mV/s. A three-electrode cell was used, which consisted of a working electrode, an Ag/AgCl electrode as the reference electrode, and a Pt coil as the counter electrode. The current density–potential curve was obtained in the range from spontaneous potential to *E* = 1.2 V. In the polarization curves (Figure 1d), a sudden increase in *I* was observed for SUS316L, indicating pitting corrosion. The pitting corrosion was not observed in the CP-Ti and TiNbTaZrMo bio-HEA AM ingots. There was no significant difference in the anodic current density between CP-Ti and AM ingots of TiNbTaZrMo bio-HEA. This indicates the superior corrosion resistance of TiNbTaZrMo bio-HEA under isotonic sodium chloride solution at 310 K (37 °C). These preliminary studies regarding the application of HEAs as metallic biomaterials, i.e., the studies on bio-HEAs, pave the way for novel applications of HEAs. The constituent elements in bio-HEAs were similar to those in RHEAs; however, the concepts of alloy design and desired mechanical and functional properties were significantly different between the bio-HEAs and RHEAs. In bio-HEAs, the biocompatibility of the constituent elements is the most important parameter for alloy design. The mechanical properties in the high-temperature region are not important for bio-HEAs, although room-temperature ductility is an important factor. The biocompatibility of bio-HEA is considered to be related to the distribution of the constituent elements [14]; however, the complexity of the constituent elements discourages the clarification of the relationship between the biocompatibility and the distribution of constituent elements. Although the high liquidus (*T*_L_) and solidus (*T*_S_) temperatures may be favorable for RHEAs, such properties are not commonly exhibited by bio-HEAs. In contrast, a significantly high *T*_L_ restricts the fabrication of bio-HEAs and increases the fabrication cost. A low *T*_L_ can overcome the limitations of the fabrication and enable the significantly more cost-effective production of bio-HEAs. Therefore, a low *T*_L_ is preferred in the development of novel bio-HEAs, from the viewpoint of materials engineering and manufacturing of metallic biomaterials. The control of the elemental distribution in the solidification microstructure and the decrease in *T*_L_ is important in the development of bio-HEAs.

The microstructure of TiNbTaZrMo bio-HEAs was investigated in detail in the as-cast state of AM ingots and in an annealed state (1273 K for 168 h), and it was discussed based on a thermodynamic calculation that was focused on the solidification and decomposition of the body-centered cubic (BCC) phase based on its miscibility gap [16]. An equiaxed dendrite structure was formed in the AM ingots. The distribution coefficient at *T*_L_ can explain the elemental distribution that led to the formation of the dual BCC phases. The elemental distribution of the ingots of bio-HEAs is one of the key factors affecting the biocompatibilities of the metallic biomaterials. The compositional dependence on the solidification microstructures and elemental distributions in the AM ingots of Ti_x_NbTaZrMo bio-HEAs has been investigated in detail in the literature [16]. The solidification microstructure analysis results in the AM ingots of Ti_x_NbTaZrMo bio-HEAs indicated that low melting temperature elements showed a tendency to be enriched in the interdendrite region in AM ingots of TiNbTaZrMo and Ti_2.6_NbTaZrMo bio-HEAs. The Ti (melting temperature, *T*_m_ = 1941 K) and Zr (*T*_m_ = 2128 K) elements were enriched in the interdendrite regions, while Mo (*T*_m_ = 2896 K) and Ta (*T*_m_ = 3290 K) were enriched in dendrite region. In this study, the solidification microstructures and elemental distributions of the ingots of the TiNbTaZr MEA and TiNbTaZrX (X = V, Mo, and W) HEAs, including the TiNbTaZrMo bio-HEA, were investigated to clarify the effect of the melting temperature of constituent elements on the solidification microstructure and elemental distributions of the ingots of the TiNbTaZrX HEAs. W and V were selected, not by biocompatibility, but by melting temperature, because the *T*_m_ of V (2183 K) is significantly lower than that of Mo (2896 K), while that of W (3695 K) is much higher than Mo. A recent review of RHEA noted that the main fabrication route of the bulk specimens of RHEAs was arc melting, arc melting and hot isostatic pressing, and arc melting and annealing (Figure 5a in [14]). In the present study, the position dependence and the formation of macroscopic solidification defects during arc melting was also investigated. Furthermore, the solidification microstructures of the AM ingot and ingots that were fabricated by cold crucible levitation melting of TiNbTaZrMo bio-HEAs were compared to clarify the cast-process dependence of the solidification microstructure. Preliminary studies of the thermodynamic calculation and the solidification microstructures in RHEAs and related MEAs [13,19,20,21] clarify the following: (1) the single BCC phase is not thermodynamically stable at room temperature in various RHEAs and related MEAs; and, (2) the distribution of constituent elements in the solidification microstructure can be predicted by a thermodynamic calculation. Yao et al. estimated the mechanical properties of RHEAs based on the distribution of constituent elements in the solidification microstructure and a simple model of solid solution strengthening [21]. A thermodynamic calculation focusing on the solidification that allowed for an estimation of *T*_L_ and *T*_S_, and the distribution coefficients at *T*_L_ were also investigated.

## 2. Materials and Methods 

TiNbTaZr MEA, TiNbTaZrMo bio-HEA, and TiNbTaZrX (X = V and W) HEAs were investigated in this study. TiNbTaZr MEA [14,17] and TiNbTaZrMo bio-HEA [14,15,16] were constructed with elements presenting high biocompatibilities. The TiNbTaZrX (X = V and W) alloys were also investigated for a comparison of the solidification microstructures among the five component TiNbTaZrX (X = V, Mo, and W) HEAs. Empirical alloy parameters for the prediction of solid solution phases in multicomponent alloys, including mixing entropy Δ*S*_mix_ [4,5,6], mixing enthalpy Δ*H*_mix_ [4,5,6], the *δ* parameter that is used to characterize the differences in atomic radii of constituent elements [4,5,6], the dimensionless *Ω* parameter, including both Δ*S*_mix_ and Δ*H*_mix_ [5,6,22], and valence electron concentration (VEC) [23], are presented in Table 1. The empirical alloy parameters of NbTaVMoW and HfNbTaZr HEAs [9,10,11,12] are also shown in Table 1 as references. The empirical alloy parameters, Δ*H*_mix_, *δ*, and *Ω*, reflect the high solid solution formation tendency, and *VEC* indicates the BCC solid solution formation tendency in TiNbTaZr MEA and TiNbTaZrX (X = V, Mo, and W) HEAs.

**Figure 2:**
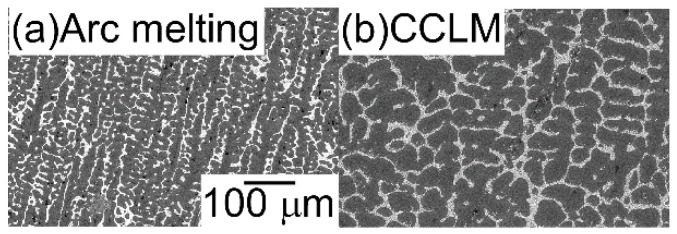
Solidification microstructure of Al_95.5_Cu_4.5_ alloy ingots prepared by arc melting (**a**), and cold crucible levitation melting (**b**). The cooling rate of arc melting and cold crucible levitation melting was estimated by the secondary dendrite arm spacing, and the microstructure was obtained from the central region of the ingots.

The AM ingots were prepared by mixing lumps of the pure elements to a total mass of approximately 20–30 g. Ti chips (purity: 3N, approximately 8 × 8 × 1 mm, Mitsuwa Chemical Co. Ltd., Japan [24]), Nb granules (purity: 3N, 2–5 mm, Mitsuwa Chemical Co. Ltd. [24]), Ta granules (purity: 3N, 2–5 mm, Mitsuwa Chemical Co. Ltd. [24]), Zr wire cuts (purity: 3N, *φ* 5 mm × 3 mm, Mitsuwa Chemical Co. Ltd. [24]), Mo shots (purity: 3N, 2 mm, Rare Metallic Co. Ltd., Japan [25]), W shots (purity: 3N, 3–5 mm, Rare Metallic Co. Ltd. [25]), and V lumps (purity: 2N, 3–10 mm, The Nilaco Co. Ltd., Japan [26]) were used. Pure element lumps presenting a small size of approximately 2–5 mm were used for the complete melting of pure-element lumps during arc melting. To achieve homogeneous distributions of the constituent elements in the ingots that were prepared by arc melting, the alloys were melted more than ten times and maintained in a liquid state for approximately 180 s during each melting event. The existence of non-molten pure element lumps was checked by optical microscopy observation of the cross-section of the AM ingots. The alloy ingots were also fabricated by cold crucible levitation melting. The cold crucible levitation melted (CCLM) ingots were obtained through induction melting under Ar flow from the AM ingots. To estimate the cooling rate of the solidification microstructure in arc melting and cold crucible levitation melting, the ingots of the Al_95.5_Cu_4.5_ alloy were prepared from the mixture of Al lumps (purity: 4N, approximately 7 × 7 × 5 mm, Mitsuwa Chemical Co. Ltd. [24]) and Cu wire cuts (purity: 4N, *φ* 2 mm × 2 mm, Mitsuwa Chemical Co. Ltd. [24]) by arc melting and cold crucible levitation melting. Secondary dendrite arm spacing (DAS) was investigated for the estimation of the cooling rate [27,28]. Figure 2 shows the solidification microstructure of Al_95.5_Cu_4.5_ alloy ingots that were prepared by arc melting and cold crucible levitation melting. The DAS in the AM ingots was much finer than that of the CCLM ingots, which indicates that the cooling rate of arc melting was much higher than that of cold crucible levitation melting. The cooling rates during arc melting and cold crucible levitation melting were roughly estimated to be on the order of 2 × 10^3^ Ks^−1^ [29] and 2 Ks^−1^, respectively.

An X-ray diffraction (XRD) analysis was performed while using a Rigaku RINT-2500 (Rigaku Corp., Tokyo, Japan) diffractometer with Cu K_α_ radiation to identify the constituent phases. The microstructures were evaluated by optical microscopy (OM), scanning electron microscopy (SEM) using SEM equipment with a W filament (JSM-5600, JEOL Ltd., Tokyo, Japan), electron probe microanalysis (EPMA), and wavelength-dispersive spectroscopy (WDS; JXA-8800R, JEOL Ltd.). The thermodynamic calculations of Ti–Nb–Ta–Zr and Ti–Nb–Ta–Zr–Mo alloys were performed with FactSage (version 7.2, FactSage, Ecole Polytechnique, Montreal, Canada) while using the thermodynamic databases for alloy systems from the Scientific Group Thermodata Europe (SGTE) 2017 [30]. Table 2 summarizes the EPMA-WDS composition analysis results of the central area of the AM ingots in TiNbTaZr MEA and TiNbTaZrX (X = V, Mo, and W) HEAs, including the TiNbTaZrMo bio-HEA. The alloy composition was obtained from wide-area EPMA-WDS analysis. From Table 2, it can be seen that there are no significant differences between the nominal alloy composition and chemical alloy composition that was evaluated by experiments in AM ingots. The value of Δ*S*_mix_ in AM ingots of TiNbTaZr MEA and TiNbTaZrX (X = V, Mo, and W) HEAs that were calculated based on the chemical composition evaluated by EPMA-WDS (Table 2) was 1.38*R* for TiNbTaZr MEA, 1.61*R* for TiNbTaZrV, 1.61*R* for TiNbTaZrMo, and TiNbTaZrW for 1.61*R*, respectively. Based on the entropy-based definition for MEA and HEA [5,6], the AM ingots of TiNbTaZr are MEAs and those of TiNbTaZrX (X = V, Mo, and W) correspond to HEAs.

## 3. Results

**Figure 3:**
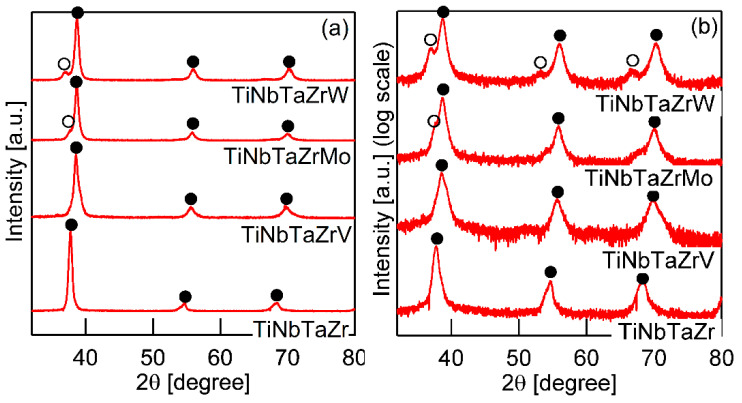
X-ray diffraction (XRD) patterns of the AM ingots of TiNbTaZr MEA and TiNbTaZrX (X = V, Mo, and W) HEAs, including the TiNbTaZrMo bio-HEA. (**a**) Linear-scale and (**b**) log-scale intensity plots.

Figure 3 shows the XRD patterns of the AM ingots for TiNbTaZr MEA, TiNbTaZrMo bio-HEA, and TiNbTaZrX (X = V and W) HEAs. A log-scale intensity plot (Figure 3b) is shown, together with a linear-scale intensity plot (Figure 3a), to enhance the minor peaks that correspond to the formation of the intermetallic compounds and those corresponding to the BCC-based ordering spots. BCC solid solution formations without intermetallic compounds were observed in the TiNbTaZr MEA, TiNbTaZrMo bio-HEA, and TiNbTaZrX (X = V and W) HEAs. Peaks corresponding to BCC-based ordering, such as the B2 structure, were not observed. The peaks of the TiNbTaZr MEA and the TiNbTaZrV HEA can be indexed to the BCC phase (as indicated by the index ●), while the formation of a dual-BCC phase that was composed of BCC-1 (indicated by the index ●) and BCC-2 (indicated by the index ○) phases was observed in the TiNbTaZrMo bio-HEA and the TiNbTaZrW HEA. In this study, the BCC-1 and BCC-2 phases are defined, as follows. The peak positions at 2*θ* of BCC-1 are higher than those of BCC-2 when the dual BCC phase forms. This indicates that the lattice constant of BCC-1 is smaller than that of BCC-2. The peaks of BCC-1 and BCC-2 was not indexed as the BCC phase of pure elements. The peaks corresponding to intermetallic compounds and/or BCC-based ordering structures, such as the B2 structure, could not be observed in the log-scale plot (Figure 3b).

**Figure 4:**
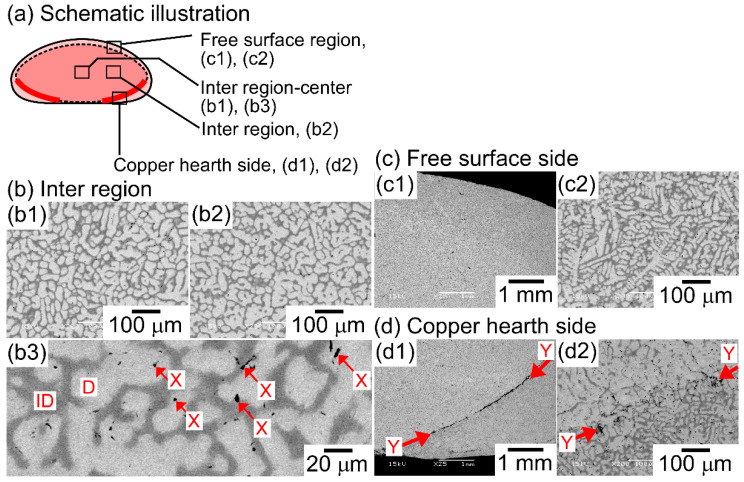
Scanning electron microscopy-back-scattering electron (SEM-BSE) images of AM ingots of TiNbTaZrMo bio-HEA focusing on position dependence. (**a**) Schematic illustration of the cross-section of the AM ingot. (**b1**) SEM-BSE image at the central region, (**b2**) SEM-BSE image at the inter-region, (**b3**) magnified SEM-BSE image at the central region showing a typical dendrite structure. (**c1**,**c2**) SEM-BSE image at the free surface side, (**d1**,**d2**) SEM-BSE image at copper hearth contacted side. Indexes D and ID indicate the dendrite and interdendrite regions. The black-contrast region indicated by index X corresponds to polishing artifacts. Index Y indicates the macroscopic solidification defect of the cold shut.

**Figure 5:**
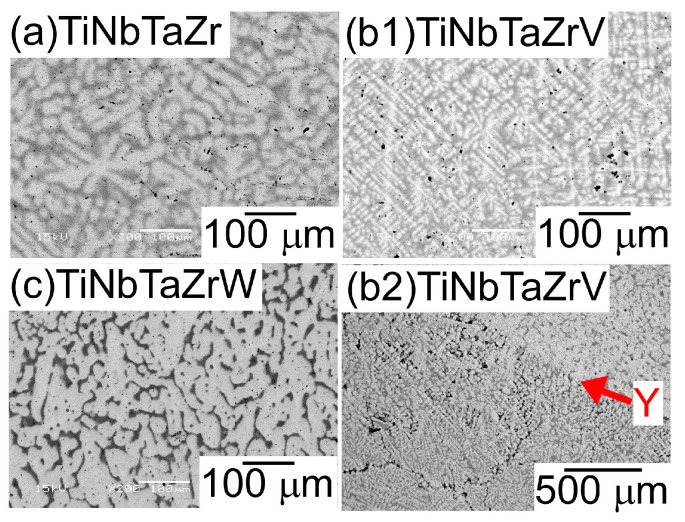
SEM-BSE images of the central region of the AM ingots of TiNbTaZr MEA and TiNbTaZrX (X = V and W) HEAs, and the region of TiNbTaZrV that made contact with the Cu hearth. (**a**) The central region of TiNbTaZr, (**b1**) the central region of TiNbTaZrV, (**b2**) the region of TiNbTaZrV that made contact with the Cu hearth, and (**c**) the central region of TiNbTaZrW. Index Y indicates the cold shut.

Figure 4 shows the SEM back-scattering electron (SEM-BSE) images of the AM ingots of the TiNbTaZr MEA, TiNbTaZrMo bio-HEA, and TiNbTaZrX (X = V and W) HEAs focusing on the position dependence of the solidification microstructures and the formation of cold shuts. Figure 4a illustrates the position of the AM ingots, where the bottom side corresponds to the side that is in contact with the Cu hearth during arc melting. The interdendrite regions (Figure 4b1,b2) show the equiaxed dendrite structure that is composed of white-gray-contrast dendrite and dark-gray-contrast interdendrite regions. Figure 4b3 is a magnified image of Figure 4b1 at the central region of the AM ingot. Indexes D and ID in Figure 4b3 indicate a typical example of a white-gray-contrast dendrite and dark-gray-contrast interdendrite. A eutectic-like structure formation and/or intermetallic compound formation in the interdendrite region could not be detected. Randomly distributed fine black-contrast regions (a typical example is indicated by index X in Figure 4b3) were observed in the AM ingots, regardless of the alloy system. These regions can be attributed as polishing artifacts to the abrasive grains from the SiC abrasive paper [31]. The formation of the equiaxed dendrite structure is also observed in the free surface region of the AM ingots, as shown in Figure 4c1,c2. Figure 4d1,d2 show the solidification microstructure in the region of the ingot that made contact with the Cu hearth (the bottom part of the photographs). One can notice the existence of a macroscopic crack with black contrast, which is indicated by index Y in Figure 4d1. The crack can be considered to correspond to the cold shut, which will be discussed in detail in the next section. Figure 4d2 show the magnified images of the cold shut, and the equiaxed dendrite structure can be observed. The formation of equiaxed dendrite structures in various regions of the AM ingots indicates that the redistribution of the constituent elements of the ingots occurred during the solidification in the arc melting process, which leads to a segregation of the elements. Figure 5 shows SEM-BSE images in the central region of AM ingots of TiNbTaZr MEA and TiNbTaZrX (X = V and W) HEAs, and the region of TiNbTaZrV that made contact with the Cu hearth. An equiaxed dendrite structure that was composed of white-gray-contrast dendrite and dark-gray-contrast interdendrite dendrite structures was observed in the AM ingots of TiNbTaZr MEA (Figure 5a), TiNbTaZrV (Figure 5b1), and TiNbTaZrW (Figure 5c) HEAs, regardless of the alloy system. The dendrite structure formation indicates the occurrence of the segregation of constituent elements. A cold shut is observed in the AM ingots of TiNbTaZr MEA and TiNbTaZrX (X = V and W) HEAs, and a typical example in the TiNbTaZrV HEA is shown in Figure 5b2, as indicated by index Y.

**Figure 6:**
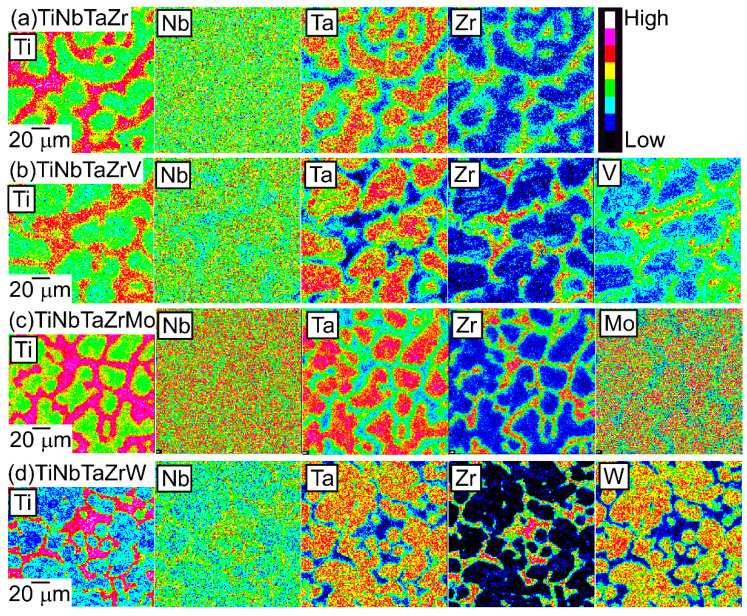
EPMA elemental maps of the central region of the AM ingots of TiNbTaZr MEA and TiNbTaZrX (X = V, Mo, and W) HEAs, including the TiNbTaZrMo bio-HEA. (**a**) TiNbTaZr, (**b**) TiNbTaZrV, (**c**) TiNbTaZrMo bio-HEA, and (**d**) TiNbTaZrW.

An EPMA-WDS analysis was performed to clarify the elemental distributions of the constituent elements in the AM ingots. Figure 6 shows the elemental maps of the central regions of AM ingots of the TiNbTaZr MEA, TiNbTaZrMo bio-HEA, and TiNbTaZrX (X = V and W) HEAs. Table 3 shows the EPMA-WDS composition analysis results of the dendrite and interdendrite regions in the central regions of the AM ingots. Irrespective of the alloy system, the following tendencies were observed in the AM ingots: (1) the interdendrite region was enriched in Ti and Zr as compared with the dendrite region; (2) the main dendrite region was enriched in Ta when compared with the interdendrite region; (3) unlike Ti, Zr, and Ta, Nb was distributed in both the dendrite and interdendrite regions; and, (4) in the TiNbTaZrX (X = V, Mo, and W) HEAs, the interdendrite regions were enriched in V in TiNbTaZrV HEA, while the interdendrite region was not enriched in Mo and W in the TiNbTaZrX (X = Mo, and W) HEAs. Both the dendrite and interdendrite regions contain all of the constituent elements, regardless of the alloy system, which indicates the formation of multicomponent BCC solid solutions. Although the chemical gradients of dendrite and interdendrite regions are important for clarifying the solidification microstructure in more detail, they were not investigated in the present study because of the limitations of the spatial resolution of the EPMA apparatus with a W-filament thermionic-emission gun being used in the present study.

The formation of dual BCC phases in the AM ingots in the TiNbTaZrMo bio-HEA is discussed in detail in the literature [15,17]; the segregation of the constituent elements leads to the formation of dendrite and interdendrite regions, resulting in the formation of dual BCC phases that are composed of BCC-1 in the dendrite region and BCC-2 in the interdendrite region. Table 4 summarized the lattice constants of BCC-1 (*a*_1_) and BCC-2 (*a*_2_), as evaluated by XRD patterns (Table 4a) and that of the dendrite (*a*_D_) and interdendrite (*a*_ID_) regions, as estimated by Vegard’s law [32] and the EPMA-WDS analysis results of AM ingots (Table 4b). The low-angle intensity peak of (110) evaluated the *a*_2_ of TiNbTaZrW, because only the (110) peak can be seen in the XRD patterns with a log-scale plot. The value of *a*_2_ in TiNbTaZr MEA, TiNbTaZrMo bio-HEA, and TiNbTaZrX (X = V and W) HEAs was not evaluated, because of the low-intensity peak of BCC-2. The value of *a*_1_ in Table 4a was similar to that of *a*_D_ in AM ingots. The application of Vegard’s law [32] in MEAs and HEAs for evaluating the lattice constants of the solid solution phase is not the established technique and it should be applied carefully in MEAs and HEAs; however, the similarity in the values between *a*_1_ and *a*_D_ indicates that the BCC phase formation can be discussed based on the lattice constants (Table 4) as well as the solidification microstructure observation by SEM (Figure 4) and EPMA-WDS (Figure 5 and Table 4). In Table 4b, *a*_ID_ was larger than *a*_D_ in the AM ingots of the TiNbTaZr MEA, TiNbTaZrMo bio-HEA, and TiNbTaZrX (X = V and W) HEAs, regardless of the alloy system. The difference in *a*_D_ and *a*_ID_, which is expressed by (*a*_ID_–*a*_D_)/*a*_D_, indicates that the difference in TiNbTaZrW HEA is much larger than those of the other alloys. The difference in *a*_D_ and *a*_ID_ in TiNbTaZrW HEA is similar to that of *a*_1_ and *a*_2_, which is expressed by (*a*_2_–*a*_1_)/*a*_1_. The correspondence implies that the formation of BCC-2 progressed as the following processes: (1) A Ta-enriched dendrite having a BCC structure was formed; (2) the ejection of the Ti and Zr elements from the dendrite region occurred, resulting in the enrichment of Ti and Zr elements in the residual liquid; (3) Ti and Zr enriched residual liquid solidified as a BCC phase, and then a Ti–Zr-enriched BCC phase was formed in the interdendrite region. The appearance of the lamellar structure and the inclusions corresponding to intermetallic compounds that formed through solidification were not detected in the interdendrite region by the microstructure analysis while using SEM (Figure 4 and Figure 5) and EPMA (Figure 6 and Table 3) in the present study. The formation of dual BCC phase structures in AM ingots of TiNbTaZrW HEA can be explained by the ejection of low-temperature elements of Ti and Zr from dendrites and the segregation at the interdendrite regions. In the AM ingots of TiNbTaZrV HEA, the Ta-enriched dendrite and the Ti–Zr–V-enriched interdendrite region are also explained by the ejection of the low melting temperature elements of Ti, Zr, and V, and the segregation of these elements at the interdendrite region.

**Figure 7:**
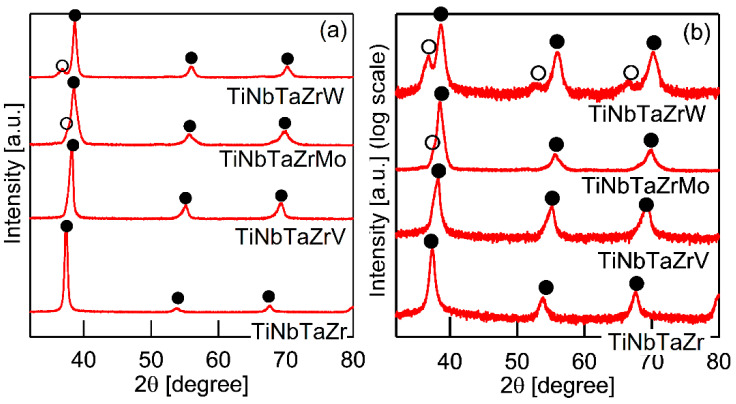
XRD patterns of cold crucible levitation melted (CCLM) ingots of TiNbTaZr MEA and TiNbTaZrX (X = V, Mo, and W) HEAs, including the TiNbTaZrMo bio-HEA. (**a**) Linear-scale, and (**b**) log-scale intensity plots.

**Figure 8:**
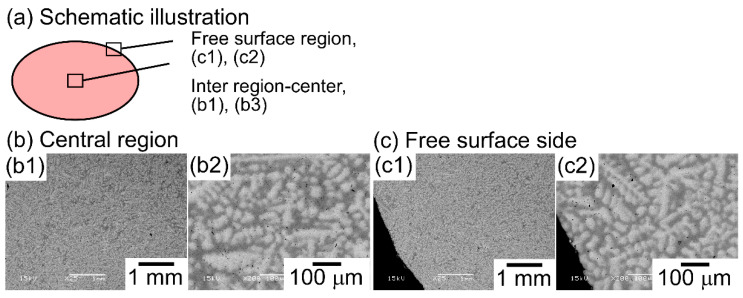
SEM-BSE images of CCLM ingots of the TiNbTaZrMo bio-HEA, focusing on the position dependence of the solidification structure. (**a**) Schematic illustration of the cross-section of the CCLM ingot. (**b1**,**b2**) SEM-BSE image at the central region, (**c1**,**c2**) SEM-BSE image at the free surface side.

**Figure 9:**
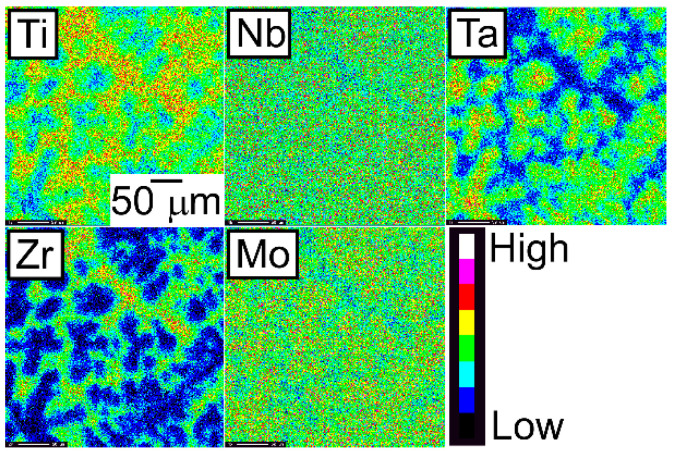
EPMA elemental maps of the central region of the CCLM ingots of the TiNbTaZrMo bio-HEA.

It is well known that the solidification microstructures in conventional alloys strongly depend on the solidification process and/or cooling rate related to the solidification rate. In this study, the influence of the casting process on the solidification microstructure was investigated, in detail, while using AM (cooling rate, ~2 × 10^3^ Ks^−1^) and CCLM ingots (cooling rate, ~2 Ks^−1^). Figure 7 shows the XRD patternsof the CCLM ingots. It is worth noting that no significant differences in the positions and intensity ratios of the peaks corresponding to BCC-1 (●) and BCC-2 (○) were observed between the ingots that were obtained by arc melting (Figure 3) and cold crucible levitation melting (Figure 7) of the TiNbTaZr MEA, TiNbTaZrMo bio-HEA, and TiNbTaZrX (X = V and W) HEAs. Figure 8 shows the solidification microstructure of the CCLM ingots of the TiNbTaZrMo bio-HEA. An equiaxed dendrite structure with a white-gray-contrast dendrite and dark-gray-contrast interdendrite regions was observed in the central region (Figure 8b) and the free surface region (Figure 8c), regardless of the position of the CCLM ingots. A eutectic-like structure and intermetallic compounds were not observed in the interdendrite region. Here, it should be noted that cold shuts were not observed in the CCLM ingots of the TiNbTaZrMo bio-HEA (Figure 8), and this tendency can also be seen in the CCLM ingots of the TiNbTaZr MEA and TiNbTaZrX (X = V and W) HEAs. Figure 9 and Table 5 show the elemental map and EPMA-WDS composition analysis results of the dendrite and interdendrite regions at the center of the CCLM ingots of the TiNbTaZrMo bio-HEA, respectively. The equiaxed dendrite structure, which consisted of a Ti–Zr-enriched interdendrite region and a Ta–Mo-enriched dendrite region, was detected in the CCLM ingots of TiNbTaZrMo bio-HEA. It should be noted that no significant differences that were related to the equiaxed dendrite structure formation and distribution of constituent elements in the solidification microstructure were observed between the AM ingots and CCLM ingots.

## 4. Discussion

**Figure 10:**
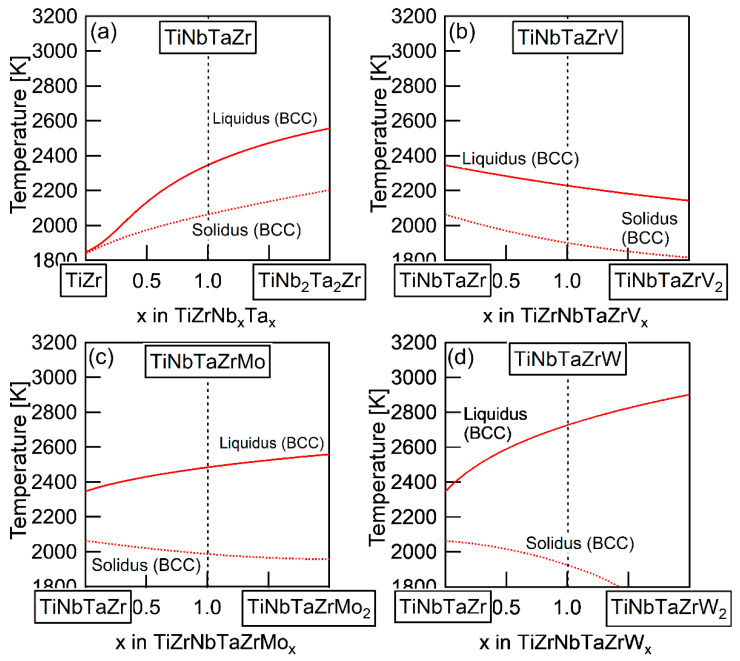
Pseudobinary phase diagrams focused on *T*_L_ and *T*_S_ of the TiNbTaZr MEA and TiNbTaZrX (X = V, Mo, and W) HEAs, including the TiNbTaZrMo bio-HEA estimated from the thermodynamic calculation considering the single BCC and single liquid phases. (**a**) TiZr–TiNb_2_Ta_2_Zr, (**b**) TiZrNbTa–TiZrNbTaV_2_, (**c**) TiZrNbTa–TiZrNbTaMo_2_, and (**d**) TiZrNbTa–TiZrNbTaW_2_.

The elemental distributions in the solidification microstructure of the AM ingot of the TiNbTaZrMo bio-HEA were explained by a thermodynamic calculation that was focused on the formation of the BCC phase through the solidification of the single liquid phase while using FactSage (version 6.4, FactSage, Ecole Polytechnique, Montreal, Canada) and SGTE2007 [17]. We discuss the elemental distributions of the dendrite and interdendrite regions in the TiNbTaZr MEA, TiNbTaZrMo bio-HEA, and TiNbTaZrX (X = V and W) HEAs based on the distribution coefficients at *T*_L_, as evaluated by the thermodynamic calculation only assuming the liquid and BCC phases using FactSage (version 7.2) and SGTE2017. The thermodynamic calculation results for the solidification in the TiNbTaZrMo bio-HEA [16] are updated in this study while using SGTE2017. Table 6 summarizes the thermodynamic calculation results of the distribution coefficients at *T*_L_ of the TiNbTaZr MEA, TiNbTaZrMo bio-HEA, and TiNbTaZrX (X = V and W) HEAs. It should be noted that no significant differences in the distribution coefficients at *T*_L_ in the TiNbTaZrMo bio-HEA were observed between the previous study (SGTE2007) [16] and this study (SGTE2017). The distribution coefficients of Ti and Zr were significantly smaller than unity, while that of Ta was significantly higher than unity, regardless of the alloy system, which indicates the formation of a Ta-rich dendrite region and the movement of Ti and Zr from the dendrite region to the residual liquid, which led to the formation of Ti–Zr-rich interdendrite regions. The distribution coefficient of V in the TiNbTaZrV HEA was smaller than unity, while those of Mo in the TiNbTaZrMo bio-HEA and W in TiNbTaZrW were larger than unity. The EPMA-WDS analysis results of the AM ingots (Figure 6 and Table 4) show the following tendency in the TiNbTaZrMo bio-HEA and the TiNbTaZrX (X = V, and W) HEA. The interdendrite region in the TiNbTaZrV HEA (Figure 6b) was enriched in V, while the interdendrite regions in the TiNbTaZrMo bio-HEA (Figure 6c) and TiNbTaZrW HEA (Figure 6d) were enriched in Mo and W, respectively. No significant differences in the elemental distributions at the dendrite and interdendrite regions were observed between AM (Figure 6c and Table 3c) and CCLM (Figure 9 and Table 5) ingots in the TiNbTaZrMo bio-HEA. The elemental distributions of the X elements in the TiNbTaZrX (X = V, Mo, and W) HEA AM ingots and those in the CCLM ingot of the TiNbTaZrMo bio-HEA can be explained by the distribution coefficients that were evaluated by the thermodynamic calculation without any discrepancy. The control of *T*_L_ is important for further development of bio-HEAs, particularly for material processing to suppress fabrication cost. A lowering of the process temperature during the casting process is significantly effective for energy savings. From an engineering viewpoint, the casting processes were strictly limited when the process temperature was over 2273 K (2000 °C), especially for the alloys that contain Ti and Zr. The significantly high *T*_L_ of RHEAs and TiNbTaZrMo bio-HEA limits the casting process, and the arc melting process is the main route in the fabrication of RHEAs [12]. A lower T_L_ was also effective in suppressing the formation of cold shuts during the arc melting process; this will be discussed in a later section. The correspondence between the solidification microstructure analysis results (Figure 3, Figure 4, Figure 5, Figure 6, Figure 7, Figure 8 and Figure 9 and Table 2, Table 3, Table 4 and Table 5) and the thermodynamic calculation results (Table 6) implies that the thermodynamic calculation is effective in predicting *T*_L_. Figure 10 shows pseudobinary phase diagrams that focus on the solidification in the TiNbTaZr MEA, TiNbTaZrMo bio-HEA, and TiNbTaZrX (X = V and W) HEAs. The pseudobinary phase diagrams were constructed while only using the Gibbs free energy of the single liquid and single BCC phases, which were used to determine the *T*_L_ and *T*_S_ of the BCC phase. *T*_L_ and *T*_S_ are represented by the solid and broken red lines in Figure 10, respectively. In the TiZr–TiNb_2_Ta_2_Zr alloy system containing the TiNbTaZr MEA (Figure 10a), *T*_L_ and *T*_S_ monotonically increased with the Nb and Ta concentrations of the TiNb*_x_*Ta*_x_*Zr (*x* = 0 − 2) alloy. *T*_L_ monotonically decreased with an increase in the V concentration of the TiNbTaZrV*_x_* alloy in the TiNbTaZr–TiNbTaZrV_2_ alloy system containing the TiNbTaZrV HEA (Figure 10b). In contrast, *T*_L_ was monotonically increased with the Mo concentration of the TiNbTaZrMo*_x_* alloy in the TiNbTaZr–TiNbTaZrMo_2_ alloy system containing the TiNbTaZrMo bio-MEA (Figure 10c). Figure 10d shows the pseudobinary phase diagram of the TiNbTaZr–TiNbTaZrW_2_ alloy system containing the TiNbTaZrW HEA. The increase in the W concentration of the TiNbTaZrW*_x_* alloy led to a significant increase in *T*_L_ and a significant decrease in *T*_S_. The changes in *T*_L_ and *T*_S_ with the concentration of the X element of the TiNbTaZrX (X = V, Mo, and W) HEA strongly depend on the X element. It was observed that an increase in the concentration of the low melting-temperature V (2183 K) led to a monotonic decrease in *T*_L_ and that increases in the concentrations of high melting-temperature Mo (2896 K) and W (3695 K) led to a monotonic increase in *T*_L_.

**Figure 11:**
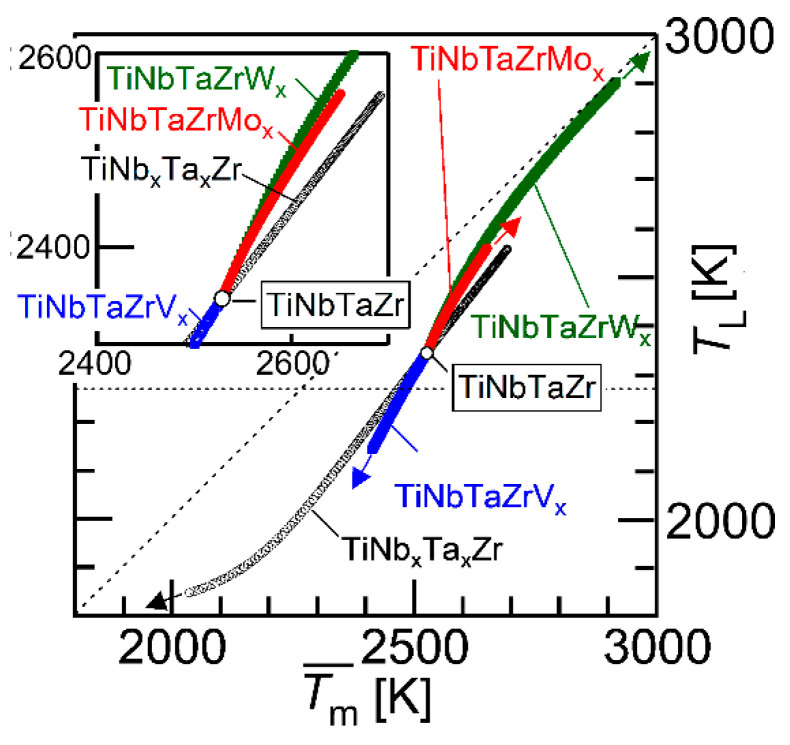
Average melting temperature ($\bar{T_{m}}$) vs. liquidus temperature estimated by thermodynamic calculation (*T*_L_) plot. The black open circle (○) indicates TiNbTaZr MEA and TiZrNb_x_Ta_x_ (0 ≤ x ≤ 2), the blue open square represents TiZrNbTaV_x_ (0 ≤ x ≤ 2), the red-filled circles correspond to TiZrNbTaMo_x_ (0 ≤ x ≤ 2), and the green-filled squares represent TiZrNbTaW_x_ (0 ≤ x ≤ 2). The inset is the magnified image.

Figure 11 shows the average melting temperature ($\bar{T_{m}}$) vs. liquidus temperature that was estimated by a thermodynamic calculation (*T*_L_) plot. The parameter ($\bar{T_{m}}$) is expressed as

(1) $$\bar{T_{m}}=\sum_{i=1}^{n}x_{i}\cdot {\left(T_{m}\right)}_{i}$$

where *x*_i_ is the mole fraction of the *i*-th element and (*T*_m_)*_i_* is the melting temperature of the *i*-th element. The black open circle (○) represents the TiNbTaZr MEA and the TiNb*_x_*Ta*_x_*Zr (*x* = 0 − 2), the blue open square represents TiNbTaZrV*_x_* (*x* = 0 − 2), the red-filled circles represent TiNbTaZrMo*_x_* (*x* = 0 − 2), and the green-filled square represents TiNbTaZrW*_x_* (*x* = 0 − 2). The arrows indicate the direction of the increase in the value of *x*. The inset is the magnified image. The *T*_L_ values of TiNb*_x_*Ta*_x_*Zr (*x* = 0 − 2), TiNbTaZrV*_x_* (*x* = 0 − 2), TiNbTaZrMo*_x_* (*x* = 0 − 2), and TiNbTaZrW*_x_* (*x* = 0 − 2) were lower than $\bar{T_{m}}$, regardless of the alloy system and the value of *x*. An increase in the value of $\bar{T_{m}}$ led to an increase in *T*_L_, regardless of the alloy system. This indicates that $\bar{T_{m}}$ can be used as a rough indicator for alloy design in decreasing *T*_L_ in TiNb*_x_*Ta*_x_*Zr and TiNbTaZrX*_x_* (X = V, Mo, and W) HEAs. The experimental measurements of *T*_L_ in RHEAs and bio-HEAs are very challenging, due to the very high *T*_L_ and reactivity of the molten state of the constituent elements; moreover, conventional solidification behavior analysis using differential thermal analysis (DTA) is not applicable. The thermodynamic calculation of *T*_L_ is helpful in the design of bio-HEAs with low *T*_L_ values.

**Figure 12:**
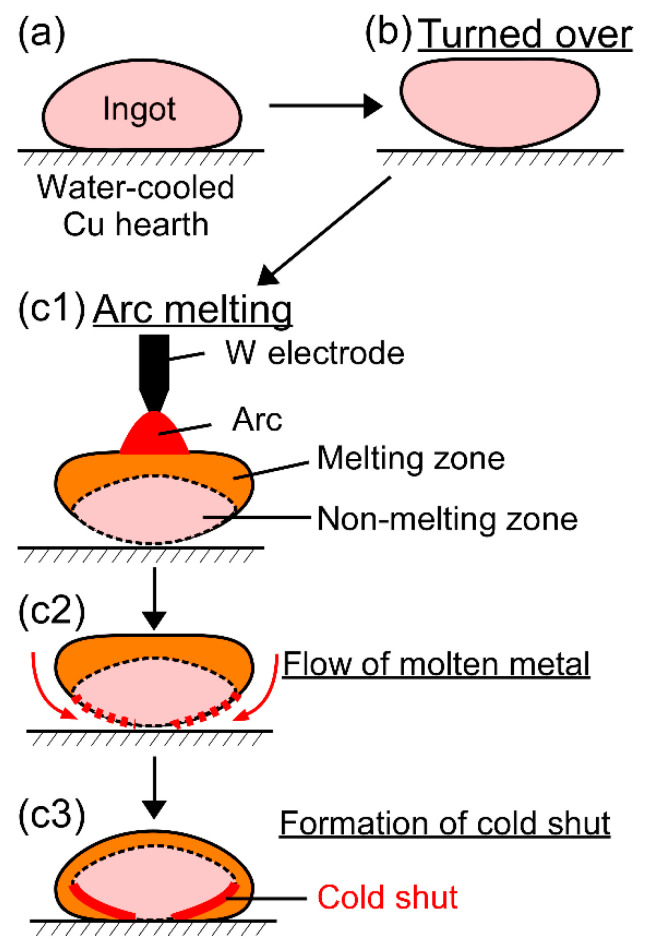
Mechanism for the formation of the cold shut during the arc melting process observed in the present study in high melting-temperature RHEAs and bio-HEAs. (**a**) Ingot on the water-cooled Cu hearth, (**b**) the ingot is turned over, and (**c1**–**c3**) formation of the cold shut during arc melting.

Cold shuts were observed in the AM ingots, as shown in Figure 4d in the TiNbTaZrMo bio-HEA and Figure 5b2 in the TiNbTaZrV HEA. Figure 12 shows a possible mechanism for the formation of a cold shut during the arc melting process that was observed in the present study. Figure 12a shows the ingot on the water-cooled Cu hearth, and Figure 12b shows the ingot after being turned over. During arc melting, the ingot in the upper part melted, while the one in the bottom part, which made contact with the water-cooled copper hearth, remained in its solid state as the non-melting zone (Figure 12c1). The flow of molten metal occurred, as shown in Figure 12c2, resulting in the formation of a cold shut during arc melting, as shown in Figure 12c3. The significantly high *T*_L_ in the TiNbTaZr MEA, TiNbTaZrMo bio-HEA, and TiNbTaZrX (X = V and W) HEAs led to the existence of the large non-melting zone in the specimens during the arc melting process, which resulted in the formation of cold shuts in the AM ingots. Based on the mechanism in Figure 12, lowering T_L_ is considered to be effective in suppressing the formation of a cold shut during the arc melting process. The formation of a cold shut was not observed in the CCLM ingots in the present study, which implied that cold crucible levitation melting processes can be effectively applied in the fabrication of bio-HEAs, and this is to be the topic of a future work.

**Figure 13:**
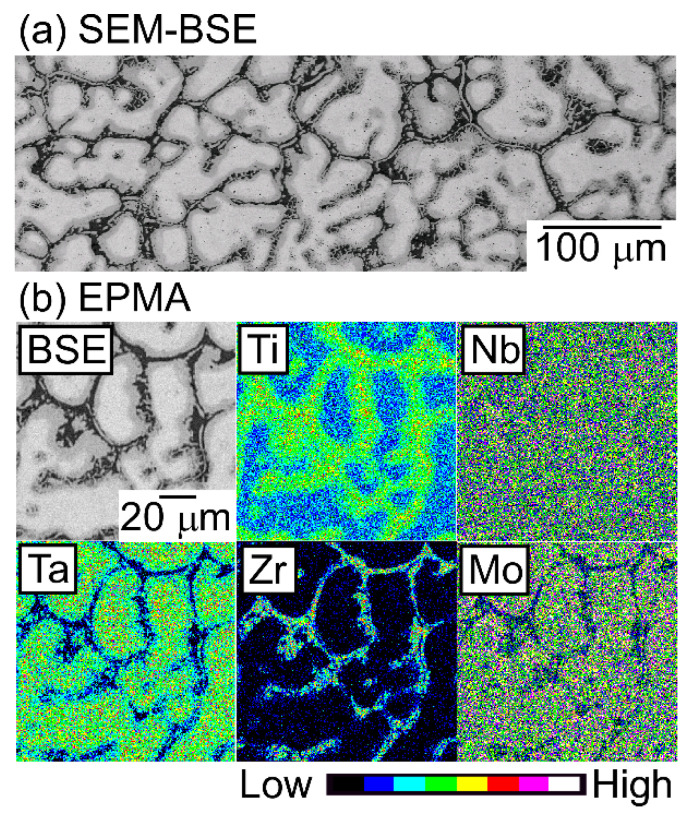
SEM-BSE image and EPMA elemental maps of the central region of the AM ingots of TiNbTaZrMo bio-HEA annealed at 1273 K (1000 °C) for 168 h (one week). (**a**) SEM-BSE image, and (**b**) EPMA elemental maps.

Finally, the annealing induced structural change in the TiNbTaZrMo bio-HEA was briefly mentioned to consider the possibility of the formation of a single BCC phase. As denoted in the literature review of RHEAs, the formation of dual BCC phases has been reported in a number of RHEAs, which is similar to the present study. One may consider that a single BCC phase can be obtained after annealing. The microstructure of annealed AM ingots is shown in Figure 13 as a typical example of the annealed structure in the TiNbTaZrMo bio-HEA. From an engineering viewpoint, the annealing temperature was strictly limited when the process temperature was over 1273 K (1000 °C), because of potential damage of the annealing furnace, the limitation of the furnace materials, and the energy cost. In the present study, the annealing temperature was set at 1273 K. Figure 13 shows an SEM-BSE image and EPMA elemental maps of the central region of the AM ingots of the TiNbTaZrMo bio-HEA annealed at 1273 K for 168 h (one week). The coarsening of the dendrite by annealing is detected in SEM-BSE images (Figure 13a). The EPMA elemental maps (Figure 13b) indicate that the Ti and Zr elements were still segregated in the interdendrite regions. In spite of the relatively long time of annealing, a single BCC phase formation was not observed in the AM ingots of the TiNbTaZrMo bio-HEA. The solidification microstructure affected the structure that was annealed at 1273 K for 168 h. The present study did not focus on the possibility of a single BCC phase structure formation. However, it can be concluded that control of the solidification microstructure and prediction of the segregation are important, not only for the ingots of bio-HEAs, but also for the annealed products of bio-HEAs.

## 5. Conclusions

The solidification microstructures of the ingots in TiNbTaZr MEA and TiNbTaZrX (X = V, Mo, and W) HEAs, including the TiNbTaZrMo bio-HEA, were investigated with a focus on their position dependence, their casting process dependence using arc melting and cold crucible levitation melting processes, the formation of cold shuts, and the distribution of the constituent elements. The distribution of constituent elements in the solidification microstructures was discussed based on their thermodynamics. The following conclusions can be drawn from the results of our study:

1. Equiaxed dendrite structures form in AM ingots of the TiNbTaZr MEA, TiNbTaZrMo bio-HEA, and TiNbTaZrX (X = V and W) HEAs, regardless of the alloy system.
2. The main dendrite phase with a BCC structure was enriched in Ta, while the interdendrite region with a BCC structure was enriched in Ti and Zr in the AM ingots of TiNbTaZr MEA, TiNbTaZrMo bio-HEA, and TiNbTaZrX (X = V and W) HEAs. It was observed that the interdendrite region was enriched in V, while Mo and W were abundant in the dendrite regions in the AM ingots of the TiNbTaZrX (X = V, Mo, and W) HEAs. The distribution coefficients during solidification, which were evaluated by thermodynamic calculations, explained the distribution of the constituent elements in the AM ingots.
3. The constituent phases in the CCLM ingots were the same as those in the AM ingots of the TiNbTaZr MEA, TiNbTaZrMo bio-HEA, and TiNbTaZrX (X = V and W) HEAs. In addition, no significant differences in the solidification microstructures related to equiaxed dendrite formation and distribution of constituent elements were observed between the AM and CCLM ingots of the TiNbTaZrMo bio-HEA, which indicated a low solidification process dependence of the solidification microstructure of the TiNbTaZrMo bio-HEA.
4. The formation of cold shuts was observed in the AM ingots, but it was not observed in the CCLM ingots of the TiNbTaZr MEA, TiNbTaZrMo bio-HEA, and TiNbTaZrX (X = V and W) HEAs.
5. Pseudobinary phase diagrams focusing on *T*_L_ and *T*_S_ of TiNbTaZr MEA and TiNbTaZrX (X = V, Mo, and W) HEAs, including the TiNbTaZrMo bio-HEA, were constructed based on thermodynamic calculations.

## Figures and Tables

**Table 1:** Empirical alloy parameters of TiNbTaZr medium-entropy alloy (MEA) and TiNbTaZrX (X = V, Mo, and W) HEAs, including TiNbTaZrMo bio-HEA, together with those of the typical refractory HEAs (RHEA).

Alloy	Δ*S*_mix_	Δ*H*_mix_	*δ*	*Ω*	VEC
NbTaVMoW	1.61*R*	−4.64	3.1	8.41	5.4
HfNbTaTiZr	1.61*R*	2.72	5.5	12.4	4.4
TiNbTaZr	1.39*R*	2.50	5.3	11.6	4.5
TiNbTaZrV	1.61*R*	0.32	6.7	101	4.6
TiNbTaZrMo	1.61*R*	−1.76	5.9	19.7	4.8
TiNbTaZrW	1.61*R*	−3.04	5.8	12.1	4.8

**Table 2:** Electron probe microanalysis-wavelength-dispersive spectroscopy (EPMA-WDS) composition analysis results of the central area of the arc-melted (AM) ingots in TiNbTaZr MEA and TiNbTaZrX (X = V, Mo, and W) HEAs, including the TiNbTaZrMo bio-HEA. The alloy composition was obtained from the wide-area scan, including dendrite and interdendrite regions.

Alloy	Ti	Nb	Ta	Zr	X
TiNbTaZr	26.2	25.7	24.1	24.1	
TiNbTaZrV	20.6	19.7	18.5	18.6	22.6
TiNbTaZrMo	21.0	19.2	21.4	19.0	19.4
TiNbTaZrW	21.8	18.1	18.7	19.6	21.8

**Table 3:** EPMA-WDS composition analysis results of the dendrite (D) and interdendrite (ID) regions in the AM ingots of TiNbTaZr MEA and TiNbTaZrX (X = V, Mo, and W) HEAs, including the TiNbTaZrMo bio-HEA. The alloy composition was obtained by point analysis. (**a**) TiNbTaZr, (**b**) TiNbTaZrV, (**c**) TiNbTaZrMo bio-HEA, and (**d**) TiNbTaZrW.

**(a)**	**Ti**	**Nb**	**Ta**	**Zr**	
D	22.9	28.7	31.2	17.2	
ID	31.4	20.0	12.2	36.5	
**(b)**	**Ti**	**Nb**	**Ta**	**Zr**	**V**
D	20.1	21.9	21.6	13.6	22.8
ID	22.4	13.6	9.2	30.1	24.7
**(c)**	**Ti**	**Nb**	**Ta**	**Zr**	**Mo**
D	22.1	19.6	20.8	16.8	20.8
ID	27.7	13.9	8.3	36.3	13.8
**(d)**	**Ti**	**Nb**	**Ta**	**Zr**	**W**
D	15.6	17.3	26.6	5.1	35.5
ID	28.0	18.9	10.8	34.2	8.2

**Table 4:** Lattice constants of lattice constants of BCC-1 and BCC-2 evaluated by the peak position in XRD patterns (**a**), and lattice constants of dendrite (*a*_D_) and interdendrite (*a*_ID_) regions estimated by Vegard’s law [32] and the EPMA-WDS analysis results (Table 4) in AM ingots (**b**).

**(a) Lattice Constants Evaluated by XRD Patterns**			
**Alloy**	***a*_1_**	***a*_2_**	**(*a*_1_ − *a*_2_)/*a*_1_ (%)**
TiNbTaZr	0.333		
TiNbTaZrV	0.327		
TiNbTaZrMo	0.332		
TiNbTaZrW	0.325	0.340	4.6
**(b) Lattice Constants Estimated by Vegard’s Law**			
**Alloy**	***a*_D_**	***a*_ID_**	**(*a*_ID_ − *a*_D_)/*a*_D_ (%)**
TiNbTaZr	0.334	0.342	2.4
TiNbTaZrV	0.328	0.333	1.4
TiNbTaZrMo	0.333	0.340	2.1
TiNbTaZrW	0.327	0.340	4.0

**Table 5:** EPMA-WDS composition analysis results of the dendrite (D) and interdendrite (ID) regions in the CCLM ingot of the TiNbTaZrMo bio-HEA. The alloy composition was obtained by point analysis.

	Ti	Nb	Ta	Zr	Mo
D	20.2	23.1	20.5	12.6	23.6
ID	26.4	15.9	7.5	34.1	16.1

**Table 6:** Distribution coefficients at *T*_L_ evaluated by thermodynamic calculation assuming liquid and BCC phases in the TiNbTaZr MEA and iNbTaZrX (X = V, Mo, and W) HEAs, including the TiNbTaZrMo bio-HEA.

Alloy	Ti	Nb	Ta	Zr	X
TiNbTaZr	0.63	1.39	1.59	0.38	
TiNbTaZrV	0.65	1.35	1.80	0.38	0.82
TiNbTaZrMo	0.53	1.21	1.61	0.24	1.41
TiNbTaZrW	0.38	1.14	1.36	0.18	1.93
